# Correction: Pertegal et al. More Than 200 Years Later: *Gluvia brunnea* sp. nov. (Solifugae, Daesiidae), a Second Species of Camel Spider from the Iberian Peninsula. *Insects* 2024, *15*, 284

**DOI:** 10.3390/insects15060452

**Published:** 2024-06-14

**Authors:** Cristian Pertegal, Pablo Barranco, Eva De Mas, Jordi Moya-Laraño

**Affiliations:** 1 Departamento de Ecología Funcional y Evolutiva, Estación Experimental de Zonas Áridas, (EEZA-CSIC) Ctra. de Sacramento s/n, La Cañada de San Urbano, 04120 Almería, Spain; demas@eeza.csic.es (E.D.M.); jordi@eeza.csic.es (J.M.-L.); 2 Departamento de Zoología, Universidad de Córdoba, Edificio C-1, Campus de Rabanales, 14071 Córdoba, Spain; 3 Departamento de Biología y Geología, CITE-IIB, Centro de Colecciones, CECOUAL, Universidad de Almería, Ctra. Sacramento, s/n, La Cañada, 04120 Almería, Spain; pbvega@ual.es

**Text Correction**

In the original publication [1], the ZooBank unique identification code (LSID—Life Science Identifier) is missing. The authors state that the scientific conclusions are unaffected. This correction was approved by the Academic Editor. The original publication has also been updated.

**Corrected Paragraph**

## 3. Results

### 3.1. Taxonomy

*Gluvia brunnea* sp. nov. Pertegal, Barranco, De Mas & Moya-Laraño.

urn:lsid:zoobank.org:pub:89EF087C-9ECF-42F8-A8C0-F3BF5CDC8063

*Holotype:* Male, Fuente del Cerezo, T.M. Berja, Sierra de Gádor, Almería, Spain, CECOUAL leg., 21 June 2021 (longitude: −2.9167199, latitude: 36.8941099; 1043 m.a.s.l.), MNCN 20.02/38113.
